# Supplementary figures and images for: Genome-wide identification and expression analysis of the Trihelix transcription factor family in potato (Solanum tuberosum L.) during development
Source: PeerJ. 2024 Nov 29;12:e18578. doi: 10.7717/peerj.18578 (PMC11610473; doi:10.7717/peerj.18578)

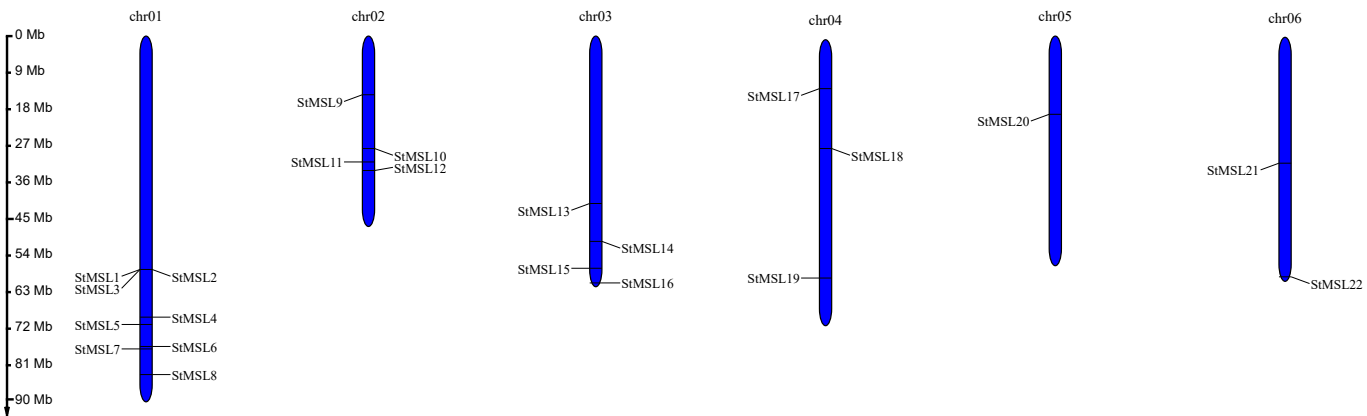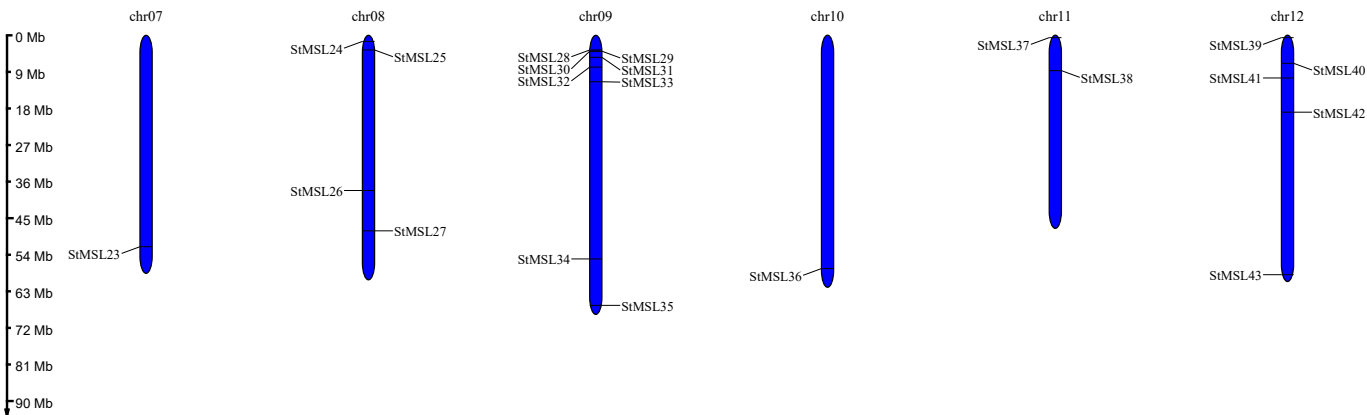

Supplement: Supplemental Information 1 [file peerj-12-18578-s001.pdf]

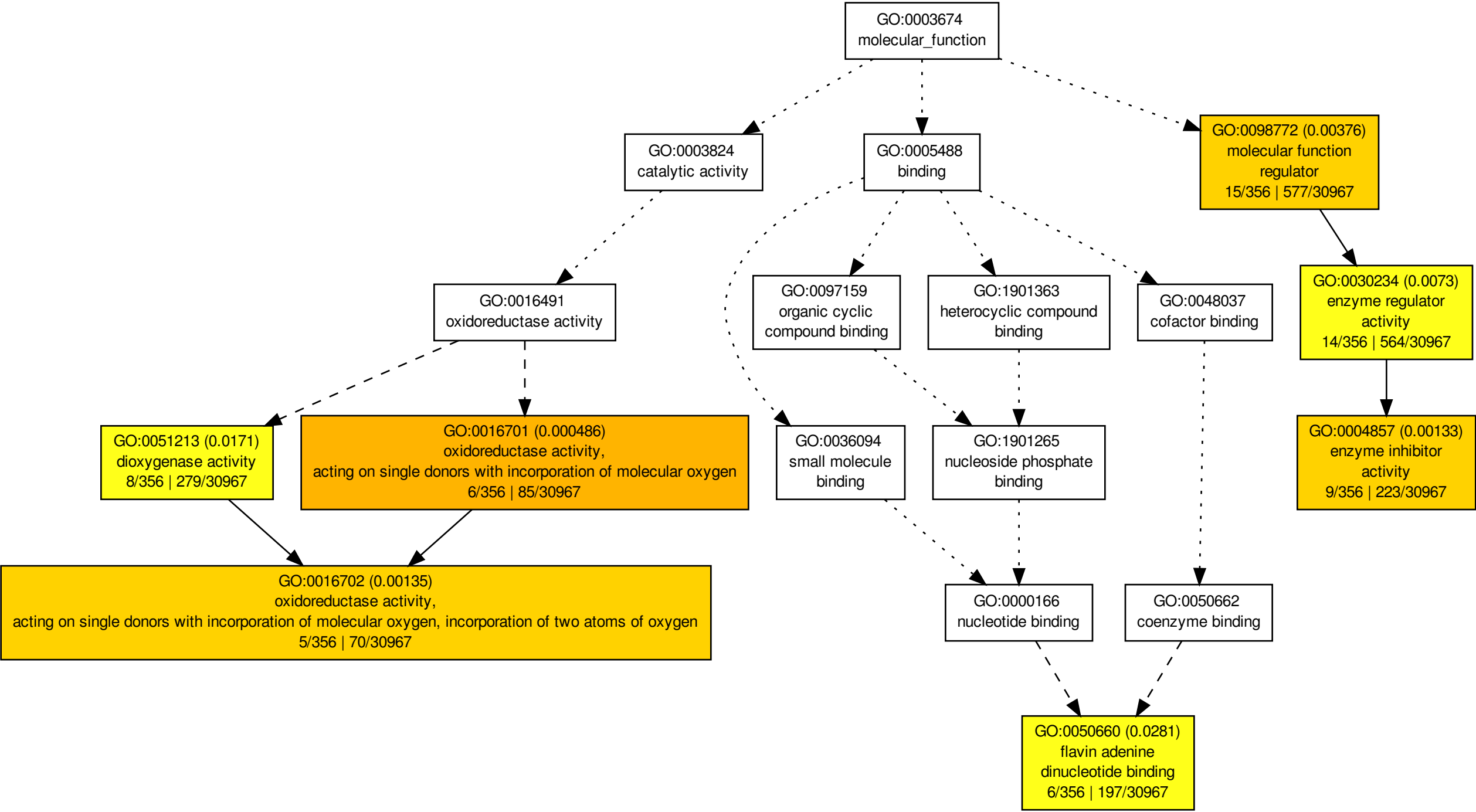

Supplement: Supplemental Information 2 [file peerj-12-18578-s002.pdf]

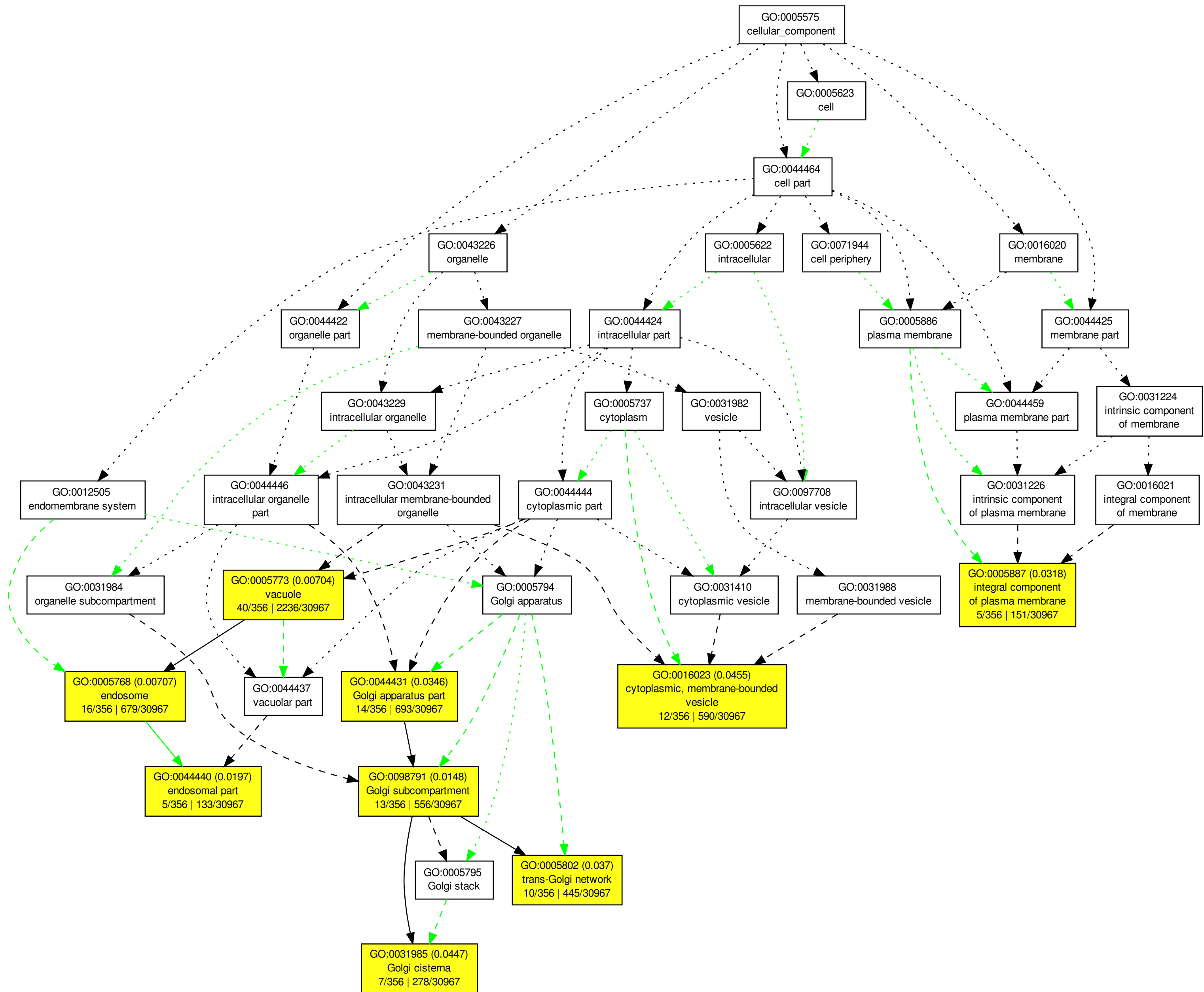

Supplement: Supplemental Information 4 [file peerj-12-18578-s004.pdf]
