# Supplementary material for: Genome-wide identification and expression analysis of the Trihelix transcription factor family in potato (Solanum tuberosum L.) during development
Source: PeerJ. 2024 Nov 29;12:e18578. doi: 10.7717/peerj.18578 (PMC11610473; doi:10.7717/peerj.18578)
Supplement: Supplemental Information 5 [file peerj-12-18578-s005.docx]

Table S1 The primers used in this study

| Gene | forward | Reverse |
| --- | --- | --- |
| StMSL4 | GGTGGCGATGGAGGTAGAAG | GAGTTCGTTCGCTGCACTTG |
| StMSL6 | CTATCCTCGCCTTGACCTTTT | CCCTCTTTCGCTTATTCTTTGA |
| StMSL14 | GCGACTGAAGGAGTTTGGGA | GCTTTCACACTGCCAAGACG |
| StMSL16 | CTTGACTTTGGCTCTACCGAT | TAGGCACATGCTGATTACCTG |
| StMSL18 | CGCCGATGATTCCAACAAAGT | GACCCCAGAGCCAAAACCAAG |
| StMSL27 | CGGCAATACCCTAGCCTCTTT | TTCCTTGAACCTGCTGTGCTT |
| StMSL30 | AAATCCCTCAACAGAACACTCA | AGCACTAACACCAGGAACAGG |
| StMSL38 | GGCATACCGAGGAACGAGAG | CCTCTGGCCTTTTCTTTTGGC |
| StMSL43 | TTCACGGCCACTCGAAGAAA | CCTACTTAGAGCACCGGCAG |
| Actin | GGGATGGAGAAGTTTGGTGGTGG | CTTCGACCAAGGGATGGTGTAGC |
