# Supplementary material for: Genome-wide identification and expression analysis of the Trihelix transcription factor family in potato (Solanum tuberosum L.) during development
Source: PeerJ. 2024 Nov 29;12:e18578. doi: 10.7717/peerj.18578 (PMC11610473; doi:10.7717/peerj.18578)
Supplement: Supplemental Information 6 [file peerj-12-18578-s006.docx]

Table S2 The location of StMSLs in the potato genome (V6.1)

| **Name** | **Gene ID** | **Chromosome** | **Start** | **End** |
| --- | --- | --- | --- | --- |
| StMSL1 | Soltu.DM.01G020620.1 | chr01 | 56407841 | 56408767 |
| StMSL2 | Soltu.DM.01G020660.1 | chr01 | 56496067 | 56498226 |
| StMSL3 | Soltu.DM.01G020710.1 | chr01 | 56602365 | 56603126 |
| StMSL4 | Soltu.DM.01G028200.1 | chr01 | 67983739 | 67988763 |
| StMSL5 | Soltu.DM.01G030180.1 | chr01 | 69910344 | 69911333 |
| StMSL6 | Soltu.DM.01G035650.1 | chr01 | 75132771 | 75135207 |
| StMSL7 | Soltu.DM.01G036430.1 | chr01 | 75910023 | 75913614 |
| StMSL8 | Soltu.DM.01G043960.1 | chr01 | 82034612 | 82040407 |
| StMSL9 | Soltu.DM.02G003400.1 | chr02 | 14203458 | 14206051 |
| StMSL10 | Soltu.DM.02G012410.1 | chr02 | 27159226 | 27163099 |
| StMSL11 | Soltu.DM.02G016000.1 | chr02 | 30518768 | 30522674 |
| StMSL12 | Soltu.DM.02G018190.1 | chr02 | 32617196 | 32621541 |
| StMSL13 | Soltu.DM.03G016380.1 | chr03 | 40497905 | 40501526 |
| StMSL14 | Soltu.DM.03G024640.1 | chr03 | 49816875 | 49829967 |
| StMSL15 | Soltu.DM.03G032480.1 | chr03 | 56353689 | 56354830 |
| StMSL16 | Soltu.DM.03G036820.1 | chr03 | 59747084 | 59748722 |
| StMSL17 | Soltu.DM.04G011000.1 | chr04 | 11716266 | 11717873 |
| StMSL18 | Soltu.DM.04G015330.1 | chr04 | 26424929 | 26435848 |
| StMSL19 | Soltu.DM.04G027080.1 | chr04 | 57664224 | 57667944 |
| StMSL20 | Soltu.DM.05G013300.1 | chr05 | 18919631 | 18920955 |
| StMSL21 | Soltu.DM.06G010060.1 | chr06 | 30609563 | 30611191 |
| StMSL22 | Soltu.DM.06G033750.1 | chr06 | 57991048 | 57992547 |
| StMSL23 | Soltu.DM.07G020890.1 | chr07 | 51283327 | 51285919 |
| StMSL24 | Soltu.DM.08G001080.1 | chr08 | 1585979 | 1588393 |
| StMSL25 | Soltu.DM.08G002920.1 | chr08 | 3526526 | 3528349 |
| StMSL26 | Soltu.DM.08G012280.1 | chr08 | 37584531 | 37586638 |
| StMSL27 | Soltu.DM.08G018560.1 | chr08 | 47342037 | 47342735 |
| StMSL28 | Soltu.DM.09G004040.1 | chr09 | 3444013 | 3447477 |
| StMSL29 | Soltu.DM.09G004470.1 | chr09 | 3892257 | 3899811 |
| StMSL30 | Soltu.DM.09G004490.1 | chr09 | 3914029 | 3916355 |
| StMSL31 | Soltu.DM.09G005630.1 | chr09 | 5212619 | 5223519 |
| StMSL32 | Soltu.DM.09G007650.1 | chr09 | 7549292 | 7551398 |
| StMSL33 | Soltu.DM.09G009310.1 | chr09 | 11136802 | 11138980 |
| StMSL34 | Soltu.DM.09G019790.1 | chr09 | 54302140 | 54303078 |
| StMSL35 | Soltu.DM.09G029350.1 | chr09 | 65442112 | 65444586 |
| StMSL36 | Soltu.DM.10G024830.1 | chr10 | 56474790 | 56477997 |
| StMSL37 | Soltu.DM.11G000160.1 | chr11 | 530027 | 532472 |
| StMSL38 | Soltu.DM.11G008900.1 | chr11 | 8565465 | 8567897 |
| StMSL39 | Soltu.DM.12G000660.1 | chr12 | 670785 | 673097 |
| StMSL40 | Soltu.DM.12G007690.1 | chr12 | 6675370 | 6678539 |
| StMSL41 | Soltu.DM.12G010580.1 | chr12 | 10383416 | 10386144 |
| StMSL42 | Soltu.DM.12G013620.1 | chr12 | 18763070 | 18763697 |
| StMSL43 | Soltu.DM.12G028480.1 | chr12 | 58159867 | 58161298 |
