# Supplementary material for: Genome-wide identification and expression analysis of the Trihelix transcription factor family in potato (Solanum tuberosum L.) during development
Source: PeerJ. 2024 Nov 29;12:e18578. doi: 10.7717/peerj.18578 (PMC11610473; doi:10.7717/peerj.18578)
Supplement: Supplemental Information 3 [file peerj-12-18578-s003.pdf]

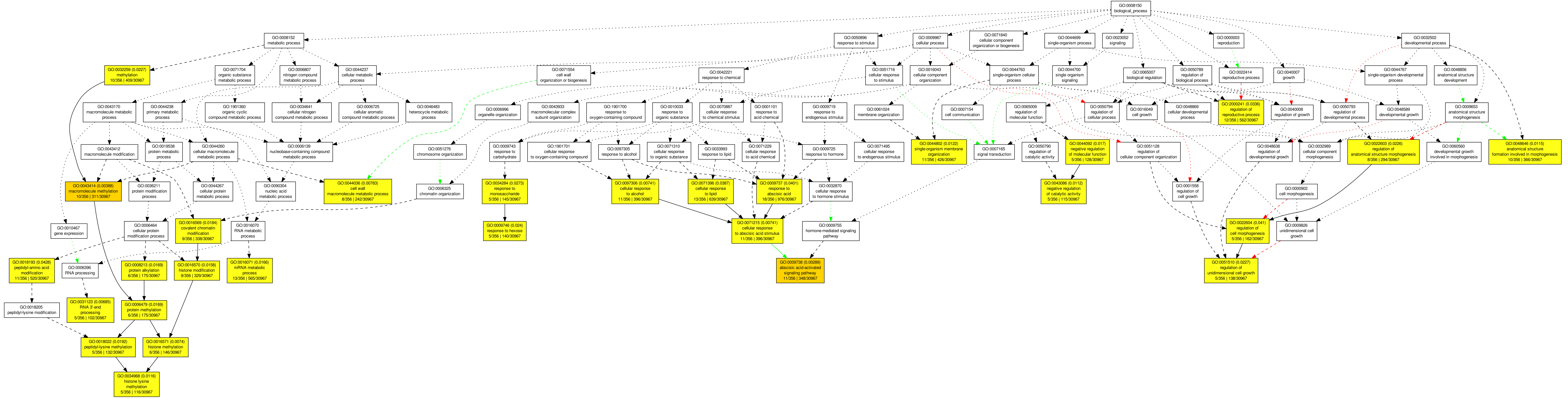
GO:0008150
biological_process
GO:0008152
metabolic process
GO:0050896
response to stimulus
GO:0009987
cellular process
GO:0071840
cellular component organization or biogenesis
GO:0044699
single-organism process
GO:0023052
signaling
GO:0000003
reproduction
GO:0032502
developmental process
GO:0032259 (0.0227)
methylation
10/356 | 409/30967
GO:0071704
organic substance metabolic process
GO:0006807
nitrogen compound metabolic process
GO:0044237
cellular metabolic process
GO:0071554
cell wall organization or biogenesis
GO:0042221
response to chemical
GO:0051716
cellular response to stimulus
GO:0016043
cellular component organization
GO:0044763
single-organism cellular process
GO:0044700
single organism signaling
GO:0065007
biological regulation
GO:0050789
regulation of biological process
GO:0022414
reproductive process
GO:0040007
growth
GO:0044767
single-organism developmental process
GO:0048856
anatomical structure development
GO:0043170
macromolecule metabolic process
GO:0044238
primary metabolic process
GO:1901360
organic cyclic compound metabolic process
GO:0034641
cellular nitrogen compound metabolic process
GO:0006725
cellular aromatic compound metabolic process
GO:0046483
heterocycle metabolic process
GO:0006996
organelle organization
GO:0043933
macromolecular complex subunit organization
GO:1901700
response to oxygen-containing compound
GO:0010033
response to organic substance
GO:0070887
cellular response to chemical stimulus
GO:0001101
response to acid chemical
GO:0009719
response to endogenous stimulus
GO:0061024
membrane organization
GO:0007154
cell communication
GO:0065009
regulation of molecular function
GO:0050794
regulation of cellular process
GO:0016049
cell growth
GO:0048869
cellular developmental process
GO:2000241 (0.0336)
regulation of reproductive process
12/356 | 562/30967
GO:0040008
regulation of growth
GO:0050793
regulation of developmental process
GO:0048589
developmental growth
GO:0009653
anatomical structure morphogenesis
GO:0043412
macromolecule modification
GO:0019538
protein metabolic process
GO:0044260
cellular macromolecule metabolic process
GO:0006139
nucleobase-containing compound metabolic process
GO:0051276
chromosome organization
GO:0009743
response to carbohydrate
GO:1901701
cellular response to oxygen-containing compound
GO:0097305
response to alcohol
GO:0071310
cellular response to organic substance
GO:0033993
response to lipid
GO:0071229
cellular response to acid chemical
GO:0009725
response to hormone
GO:0071495
cellular response to endogenous stimulus
GO:0044802 (0.0122)
single-organism membrane organization
11/356 | 426/30967
GO:0007165
signal transduction
GO:0050790
regulation of catalytic activity
GO:0044092 (0.017)
negative regulation of molecular function
5/356 | 128/30967
GO:0051128
regulation of cellular component organization
GO:0048638
regulation of developmental growth
GO:0032989
cellular component morphogenesis
GO:0022603 (0.0226)
regulation of anatomical structure morphogenesis
8/356 | 294/30967
GO:0060560
developmental growth involved in morphogenesis
GO:0048646 (0.0115)
anatomical structure formation involved in morphogenesis
10/356 | 366/30967
GO:0043414 (0.00388)
macromolecule methylation
10/356 | 311/30967
GO:0036211
protein modification process
GO:0044267
cellular protein metabolic process
GO:0090304
nucleic acid metabolic process
GO:0044036 (0.00783)
cell wall macromolecule metabolic process
8/356 | 242/30967
GO:0006325
chromatin organization
GO:0034284 (0.0273)
response to monosaccharide
5/356 | 145/30967
GO:0097306 (0.00741)
cellular response to alcohol
11/356 | 396/30967
GO:0071396 (0.0387)
cellular response to lipid
13/356 | 639/30967
GO:0009737 (0.0401)
response to abscisic acid
18/356 | 976/30967
GO:0032870
cellular response to hormone stimulus
GO:0043086 (0.0112)
negative regulation of catalytic activity
5/356 | 115/30967
GO:0001558
regulation of cell growth
GO:0000902
cell morphogenesis
GO:0010467
gene expression
GO:0006464
cellular protein modification process
GO:0016569 (0.0184)
covalent chromatin modification
9/356 | 338/30967
GO:0016070
RNA metabolic process
GO:0009746 (0.024)
response to hexose
5/356 | 140/30967
GO:0071215 (0.00741)
cellular response to abscisic acid stimulus
11/356 | 396/30967
GO:0009755
hormone-mediated signaling pathway
GO:0022604 (0.041)
regulation of cell morphogenesis
5/356 | 162/30967
GO:0009826
unidimensional cell growth
GO:0018193 (0.0428)
peptidyl-amino acid modification
11/356 | 520/30967
GO:0006396
RNA processing
GO:0008213 (0.0169)
protein alkylation
6/356 | 175/30967
GO:0016570 (0.0158)
histone modification
9/356 | 329/30967
GO:0016071 (0.0166)
mRNA metabolic process
13/356 | 565/30967
GO:0009738 (0.00289)
abscisic acid-activated signaling pathway
11/356 | 348/30967
GO:0051510 (0.0227)
regulation of unidimensional cell growth
5/356 | 138/30967
GO:0018205
peptidyl-lysine modification
GO:0031123 (0.00685)
RNA 3'-end processing
5/356 | 102/30967
GO:0006479 (0.0169)
protein methylation
6/356 | 175/30967
GO:0018022 (0.0192)
peptidyl-lysine methylation
5/356 | 132/30967
GO:0016571 (0.0074)
histone methylation
6/356 | 146/30967
GO:0034968 (0.0116)
histone lysine methylation
5/356 | 116/30967
